# Women with Endometriosis—Who Is at Risk for Complications Associated with Pregnancy and Childbirth? A Retrospective Case–Control Study

**DOI:** 10.3390/jcm13020414

**Published:** 2024-01-11

**Authors:** Teresa Mira Gruber, Laura Ortlieb, Wolfgang Henrich, Sylvia Mechsner

**Affiliations:** 1 Department of Obstetrics, Charité—Universitätsmedizin Berlin, Corporate Member of Freie Universität Berlin and Humboldt-Universität zu Berlin, Augustenburger Platz 1, 13353 Berlin, Germany; 2 Endometriosis Centre Charité, Department of Gynaecology, Charité—Universitätsmedizin Berlin, Corporate Member of Freie Universität Berlin and Humboldt-Universität zu Berlin, Augustenburger Platz 1, 13353 Berlin, Germany

**Keywords:** endometriosis, deep infiltrating endometriosis, adenomyosis, pregnancy outcome, preterm delivery, caesarean delivery, placenta praevia

## Abstract

Women with endometriosis (EM), particularly the manifestations of adenomyosis (AM) and deep infiltrating endometriosis (DIE), suffer from pain and sterility. DIE also appears with several specific obstetric complications. To determine the risk profile, we designed a retrospective case–control study. Primary outcomes were defined as the risk of preterm birth and caesarean delivery (CD). Primiparous singleton pregnancies in women with DIE were compared with controls without EM. We matched for mode of conception and maternal age. A total of 41 women diagnosed with DIE and 164 controls were recruited. A total of 92.7% of the cases were also diagnosed with AM. Preterm birth occurred in 12.2% of cases and in 6.7% of controls. The difference was not statistically significant (OR: 1.932; 95% CI: 0.632–5.907). The rate of CD was similar in both groups. Remarkably, placental implantation disorders in the form of placenta praevia were eight times more frequent in women with DIE (9.8%) than in controls (1.2%, OR: 8.757; 95% CI: 1.545–49.614). Neonatal outcome was similar in both groups. Four out of fourteen cases reported abdominal wall endometriosis after CD. Women with DIE/AM and with placenta praevia are at risk of bleeding complications. After CD, they can develop abdominal wall EM. We therefore suggest evaluating the birth mode in each woman with DIE/AM.

## 1. Introduction

Endometriosis (EM) is a common disease in women, and its main symptoms are chronic pain and the unfulfilled desire to have children [1,2,3]. In addition, EM appears to be associated with specific risks for pregnant women and the unborn child. For example, an Italian meta-analysis from 2017 pooled the results of 24 observational studies with a total of 52,111 women with EM and 1,872,003 controls without EM. According to Zullo et al., women with EM had a significantly higher incidence of miscarriages, preterm birth, placenta praevia and caesarean delivery (CD), and gave birth to significantly more low-birth-weight babies than the control group [4]. However, the quality of the studies was variable, and most of the women with EM in the study gave birth without complications. The newborns were term with normal weight [4]. Despite this, there have been case reports of severe complications, such as a rectal injury during labour (Menzlova et al., 2014 [5]). The frequency of such catastrophic events, which are presumably rare, is not known because studies with appropriate case numbers are lacking. It is also important to note that in almost all the previous trials to date, there are two major confounding factors. One: assisted reproductive technology (ART). Women with EM are more likely to seek reproductive assistance [6]. Artificial insemination in the form of in vitro fertilisation (IVF) or intracytoplasmatic sperm injection (ICSI) is associated with pre-eclampsia, premature placental abruption, placenta praevia and CD. Notably, it appears that women with EM who have undergone IVF have an even higher risk of placenta praevia [7]. Two: maternal age. It is a risk factor for several obstetric complications, including gestational hypertension and CD [8]. The heterogeneous management of confounders is probably one of the reasons why preliminary studies have not yet shown comparable results. 

Who are the women at risk?

We believe that DIE combined with AM leads to an increased risk of pregnancy and birth complications, as the uterus and its immediate surroundings are the focus of the disease. We experience that deep infiltrating endometriosis (DIE) without concomitant adenomyosis (AM) is rare. Lazzeri et al. made a similar observation in an Italian study [9]. Kunz et al. confirmed the strong association between AM and EM in extensive radiological examinations of women with an unfulfilled desire to have children [10]. 

In this study, our aim was to determine the risk profile of women with DIE in combination with AM, as we expect them to have the highest rate of complications. 

Our primary outcomes were defined as the risk of preterm birth and CD. Secondary outcomes were other complications during pregnancy and delivery, as well as neonatal outcome. We adjusted for the two main confounders, namely ART and maternal age.

## 2. Materials and Methods

We compared pregnancies in women with DIE with pregnancies in women without EM in a matched retrospective case–control study. We followed the Strengthening the Reporting of Observational Studies in Epidemiology (STROBE) statement [11] (Supplementary Data S1). 

Objectives

The primary endpoints of the study were the rates of CD and preterm birth. 

As secondary endpoints, we evaluated the risk of placental abnormalities, hospitalisation during pregnancy, hypertensive pregnancy disorders, foetal presentation, rare complications, neonatal outcome and abdominal wall EM. 

Recruitment

Participants were recruited at the certified Endometriosis Centre of the Charité, Berlin (hereafter Endometriosis Centre). Every year, more than 1000 women with EM are treated and around 150 operations are performed (Endometriosezentrum: Fächerverbund Frauenheilkunde-Charité—Universitätsmedizin Berlin (charite.de)). 

Controls were recruited from the patient population at Charité Berlin, a tertiary care hospital.

Inclusion criteria

Minimum age of 18 years, postal address in Germany, written consent to participate and return of a completed questionnaire. We defined cases as primary singleton pregnancies that resulted in the birth of a live or dead infant with a birth weight greater than 500 g. For the diagnosis of EM and AM, we expected to have at least one documented consultation with an interview, clinical examination and ultrasound by Prof. Mechnser (between 1 January 2015 and 31 December 2019) and a diagnosis of ENZIAN classification ABC or FI.

Exclusion criteria

Women with an unclear diagnosis of DIE, other serious maternal illness prior to pregnancy, uterine surgery with opening of the uterine cavity and multiple pregnancies were excluded (Supplementary Data S4). 

Data collection and statistical methods

A questionnaire was used to collect pregnancy-related data (Supplementary Data S3). Each woman who visited the Endometriosis Centre with a diagnosis of DIE during this period was contacted once by post.

Data collection for the control group was conducted retrospectively from electronic clinic records and included patient data from 2015 to 2019.

Cases and controls were matched in a ratio of one to four based on maternal age (+/− two years) and ART use IVF and ICSI. Univariable logistic regression models were used to analyse the primary endpoints (preterm birth and CD). The *p*-values of the primary analyses were adjusted according to Bonferroni–Holm, as two primary endpoints were examined.

## 3. Results

In our retrospective case–control study, we compared primiparous singleton pregnancies in 41 women with DIE and 164 women without EM.

Cases

**Figure 1:**
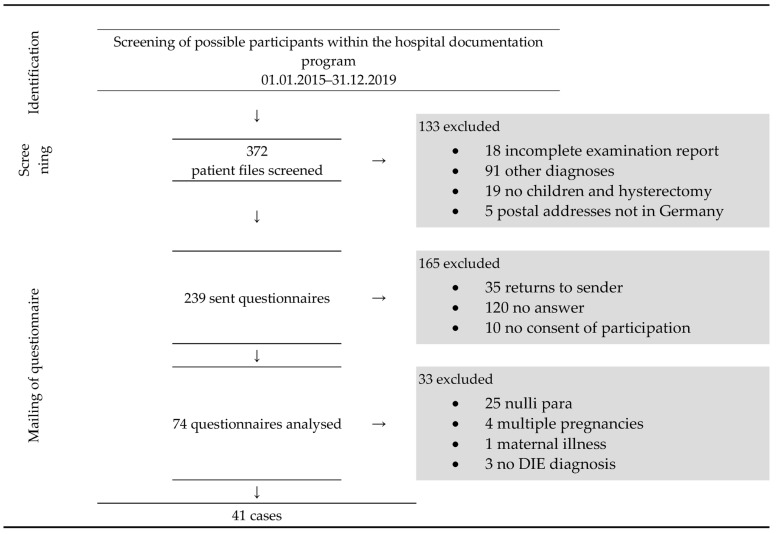
Flow chart of recruitment of cases. DIE: deep infiltrating endometriosis.

A questionnaire was mailed to 239 eligible participants. After screening, 41 primary singleton pregnancies in women were included. Figure 1 summarises the recruitment.

The median age of the participants at the time of the diagnosis was 28 years. All the participants had undergone surgery for DIE, 61% of the participants at least once prior to the case pregnancy (Table 1). A total of 92.7% were also diagnosed with AM, and 73.2% were diagnosed with rASRM III-IV (revised Classification of the American Society for Reproductive Medicine for endometriosis) (Table 2). The median age at case pregnancy was 29 years. For 93% of the participants, the case pregnancy was their first pregnancy. Twenty-nine per cent gave birth to other children after the case pregnancy.

Controls

**Figure 2:**
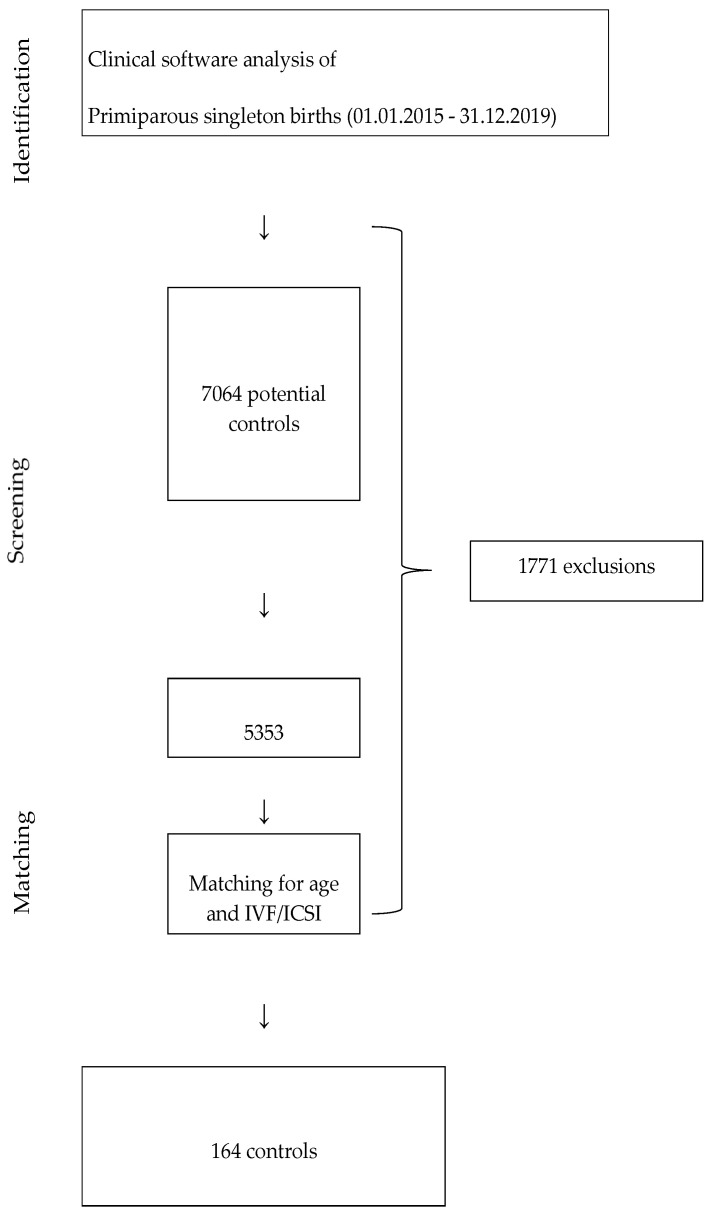
Selection of controls. IVF/ICSI: in vitro fertilisation/intracytoplasmatic sperm injection.

Figure 2 summarises the control selection process. Maternal illnesses as exclusion criteria are listed in Supplementary Data S4.

The 41 participants and the 5,353 potential controls had the same median age (29 years). A total of 31.7% of the participants had become pregnant by IVF or ICSI while only 4.2% of the 5353 potential controls had (*p* < 0.001). The control group was selected from the 5353 potential controls by exact matching on age (+/− two years) and mode of conception. Table 3 summarises the results of the matching process.

### 3.1. Preterm Birth

The median gestational age at birth was 40 + 0 weeks in the cases and 39 + 5 weeks in the controls. Five of forty-one participants with DIE (12.2%) and eleven of one hundred and sixty-four controls (6.7%) gave birth before 37 + 0 weeks gestation. The OR for preterm birth in women with DIE was 1.932 but was not statistically significant (95% CI: 0.632–5.907). Two of forty-one cases (4.9%) and six of one hundred and sixty-four controls (3.7%) gave birth before 34 + 0. In the case group, three of the five preterm births (60%) were due to placenta praevia with haemorrhage. Other reasons were preterm premature rupture of membranes (PPROM) and cervical insufficiency. In the control group, six preterm births were due to PPROM, four were due to hypertensive disorders and one was due to cervical insufficiency.

### 3.2. Birth Mode

Regarding birth modes, 14 of 41 cases (34.1%) and 52 of 164 controls (31.7%) gave birth by CD (OR: 1117, CI: 0.541–2.305). In both groups, approximately one-third were primary and two-thirds were secondary CD. Three participants reported that they had been advised to have a CD because of previous surgery for EM/AM. As an incidental finding, four out of fourteen participants (29%) who had a CD reported the development of abdominal wall EM at the caesarean scar. Unfortunately, no further details are available, as abdominal wall EM was not part of the study design.

### 3.3. Secondary Endpoints

As secondary endpoints, we evaluated the risk of placental abnormalities, hospitalisation during pregnancy, hypertensive disorders, including pre-eclampsia (PE) foetal presentation, rare complications, and neonatal outcome (Table 4).

#### 3.3.1. Placenta Praevia

Four cases out of forty-one women (9.8%) had a placenta praevia in the case pregnancy (*p* = 0.004) with an OR of 8.757 (Table 4).

#### 3.3.2. Hospitalisation during Pregnancy

Case patients reported more hospital admission during pregnancy than controls (22% vs. 13.4%). Cases with an inpatient stay during pregnancy spent a median of five nights in hospital, whereas controls with an inpatient stay during pregnancy spent a median of only three nights. The differences were not significant (Table 4).

#### 3.3.3. Other Complications and Foetal Presentation

We found no differences in hypertensive disorders, including preeclampsia (PE), PPROM, placental abruption or foetal presentation between women with DIE and controls without EM. Foetal presentation was similar in both groups (Table 4).

#### 3.3.4. Rare Severe Complications

Uterine rupture, spontaneous intestinal perforation, spontaneous hematoperitoneum during pregnancy or placenta increta or percreta did not occur in our cohort.

#### 3.3.5. Neonatal Outcome

Neonatal outcome was similar in both groups (Table 5).

The median umbilical artery pH in both groups was above 7.20. The proportion of children with umbilical artery pH below 7.10 did not differ significantly between the groups. Six of the forty-one case patients did not report umbilical artery pH, while only one control had no documented umbilical artery pH. The APGAR (acronym for appearance, pulse, grimace, activation and respiration, classification for postnatal adaptation) values of the neonates in both groups five and ten minutes after birth were not different. The neonates from both groups had a median birth weight of 3330 g and 3323 g, respectively, and a median weight slightly below the 50th percentile for gestational age and sex. The proportion of infants with a birth weight below the 10th percentile or above the 95th percentile was similar in both groups (Table 5).

## 4. Discussion

Our results show that preterm birth occurred in 12.2% of the women with DIE and in 6.7% of the controls, although the difference was not statistically significant (OR: 1.932; 95% CI: 0.632–5.907). The rate of CD was similar in both groups. Despite that, placenta praevia was eight times more common in primiparous women with DIE (9.8%) than in controls (1.2%, OR: 8.757; 95% CI: 1.545–49.614). After CD, four out of fourteen (28.6%) cases reported suffering from abdominal wall EM. Neonatal outcome was similar in both groups.

In our cohort, DIE did not occur as a single diagnosis. Women diagnosed with DIE were also diagnosed with AM and rASRM III-IV in almost all our cases. Therefore, the effects of AM and DIE on pregnancy and delivery cannot be distinguished based on our data. The pathophysiological mechanisms leading to specific obstetric complications associated with EM are unclear and are most likely to be multiple.

### 4.1. Women with Complex EM

According to ENZIAN, AM is classified as a manifestation of DIE [12]. We postulate and follow the theory of Leyendecker et al. that there is one single origin of EM, DIE and AM, namely *archimetrosis* [13]. In his theory, the uterus is the primary source of progression of the disease. Uterine hyperperistalsis leads to chronic tissue injury processes in the junctional zone. As a result, repair programmes are upregulated and stem cells are activated. Within the myometrium, these mechanisms lead to structural and functional changes [14]. Activated stem cells can also leave their niche and migrate into the myometrium or through the fallopian tubes into the pelvic cavity. Endometrial tissue can thus disperse and form new endometriosis lesions [15,16].

We postulate further that the greater the architectural changes within the uterus, the greater the influence on the course of pregnancy. In particular, patients with DIE and placenta praevia have been shown to have greater blood loss during CD than patients without DIE [17]. The differentiated investigation of these pathophysiological effects is a major challenge for future studies.

### 4.2. Preterm Birth

Our participants were almost twice as likely to have a preterm birth (12.2%) than controls without EM (6.7%), but this difference was not statistically significant (*p* = 0.241). A possible explanation for the increased rate of preterm birth is the frequency of placental abnormalities in women with DIE: three case patients reported placenta praevia with haemorrhage as the cause of preterm birth. Only one woman with placenta previa did not report bleeding complications prior to CD.

### 4.3. Mode of Delivery

Interestingly, we did not find an increased rate of CD in women with DIE compared with controls without EM (34.1% vs. 31.7%; *p* = 0.765), despite the number of women with placenta praevia. In previous studies, CD rates in patients with DIE ranged from 32.3% to 68.3% [18]. Interestingly, the CD rates in the control groups of the respective studies also differed significantly from study to study: 21% [19] vs. 43.3% [18]. Cases and controls in our study differed significantly regarding the year in which the cases’ pregnancies ended (cases 1988–2020, controls 2015–2019) and the hospitals in which they gave birth (cases in various hospitals throughout Germany, controls at Charité Universitätsmedizin Berlin). This may have biased our results for CD: Cases who gave birth earlier may have had a lower rate of CD as this was the general practice in obstetrics at that time. This may also explain the relatively low rate of CD in our case group.

In a study by Exacoustos et al., women with DIE had significantly more complications during CD, such as hysterectomies, hemoperitoneum and urinary bladder injury than women without EM [18]. The same finding was reported in a French observational study by Thomin et al. Unfortunately, the study did not include a control group. Seventy-two pregnancies in women with DIE were analysed and there were significantly more complications with a CD than with a vaginal birth [20]. One possible explanation is that women with complex EM often have adhesions between the female genital organs, the abdominal wall and/or the bowel. Bleeding complications during CD have previously been reported in women with posterior extrauterine adhesions. Bleeding could be reduced by avoiding uterine extortion during surgery [17].

No one in the case group reported a major complication during vaginal delivery. Exacoustos et al. and Nirgianakis et al. also reported comparable complication rates of vaginal deliveries in women with DIE and without EM [18,19].

### 4.4. Abdominal Wall Endometriosis Post CD

Almost a third of the case patients in our case–control study who gave birth by CD (four out of fourteen) reported the development of abdominal wall EM after CD. This was not reported by any of the women in the case group who gave birth vaginally or in the control group. The deliveries of the four women with abdominal wall EM included two primary and two secondary CDs. All operations were performed using the Misgav Ladach technique. The four women were all diagnosed with a complex and extensive form of EM and had undergone multiple operations for this diagnosis prior to CD.

Abdominal wall EM is defined as endometriotic lesions infiltrating the subcutaneous fat, the rectus muscles or the skin. These develop months or years after a CD but have also been described after vaginal tears during childbirth [21,22]. The women suffer from cyclic pain and local swelling in the scar region. In rare cases, there may be cyclic bleeding from the scar [23]. Diagnosis is often difficult as the symptoms are misinterpreted and imaging by ultrasound and/or MRI can be difficult. Treatment includes the surgical excision of the affected tissue as well as hormone therapy as a follow-up. Recurrence rates can be as low as 1% [24] and as high as 13% [25].

Little is known about the general epidemiology. Ding et al. retrospectively analysed the medical records of all 7478 women who underwent surgery for EM at Fudan University Hospital in Shanghai between 2003 and 2011. A total of 3.04% of the women (227) developed abdominal wall EM [26].

CD in a patient’s history seems to be the most common risk factor for the development of abdominal wall EM [23].

Translocation of activated stem cells from the junctional zone to the abdominal wall may cause the development. The ectopic lesions are always composed of epithelial and stroma cells as well as myometrial-like smooth muscle cells. These findings support the hypothesis of a disease based on translocated uterine stem cells.

Stem cells can translocate during surgery and invade the skin, the soft tissues or the muscles of the abdominal wall. Perhaps the higher amount of activated uterine stem cells in patients with AM is a possible explanation for the high number of secondary abdominal wall EM.

The indication for CD in women with AM and DIE should be strict to avoid serious complications.

### 4.5. Placenta Praevia

In our case–control study, cases with DIE in primiparous singleton pregnancies had a significantly higher incidence of placenta praevia than controls without EM (9.8% vs. 1.2%; OR = 8.757; 95% CI: 1.545–49.614; *p* = 0.004). Correspondingly, previous studies have found increased rates of placenta praevia in women with DIE [18,19,27,28]. AM-associated disruption of the junctional zone and the inner myometrium may lead to placental implantation disorders [29,30,31,32]. In particular, the DIE-associated involvement of the posterior compartment appears to increase the risk of placenta praevia. An association between AM of the posterior uterine wall and placenta praevia has been demonstrated and posterior adhesions may be a risk factor for bleeding complications [33].

As the primary endpoints of this case–control study were preterm birth and CD rate, the results on placenta praevia are exploratory and would need to be confirmed in the future.

### 4.6. Neonatal Outcome

There is evidence in the literature that neonates born to mothers with AM are significantly lighter, more likely to be less than 2500 g and 1500 g and to be below the 10th weight percentile compared to neonates of mothers without EM [18,19,27,34]. We were not able to reproduce these findings. The median birth weight, the median weight percentile, the Apgar scores and the rate of infants with an umbilical artery pH below 7.20 or 7.10 were similar in the cases and the controls. The distributions of the foetal presentation at birth and the sex of the neonates were equal.

### 4.7. Limitations

Of the 205 women who received the questionnaire, 74 responded with signed informed consent, giving a response rate of 36%. The questionnaire and participant information were written in German. Potential participants with little or no knowledge of German were not able to participate in the study. We cannot exclude the possibility of confounding in both directions due to the frequent non-participation of patients with particularly many or few complications during pregnancy. We included 41 women with a first diagnosis of DIE before or after the case pregnancy and women with or without surgery for DIE before the case pregnancy. Data on cases and controls were collected retrospectively. This may have biased the results due to misattribution and recall bias. EM is a very common and under-diagnosed condition, and sonographic diagnosis in pregnancy is particularly difficult. Twelve potential controls were excluded because pre-existing EM or AM was documented. However, EM or AM could not be ruled out with certainty in the included controls. This could lead to an underestimation of the presumed increased risk in the case patients, i.e., a confounding in the direction of the null hypothesis. There could be a distortion of the results because the controls were recruited within a tertiary care hospital, e.g., for patients with gestational diabetes, which could not be completely excluded. This could lead to an underestimation of the presumed increased risk in women with DIE, i.e., a confounding in the direction of the null hypothesis. In general, the pregnancies of the controls did not differ much from the results of the federal evaluation of the IQTIG for all hospital births in Germany in 2019 for the endpoints analysed [35]. We assume that our control group is therefore representative of primiparous singleton births in Germany.

## 5. Conclusions

Most patients with DIE appear to have uncomplicated vaginal births with a good outcome for both mother and child. A number of women may have rare dangerous complications, though. CD in women with DIE is more difficult and associated with more complications than CD in women without EM.

Women with DIE/AM have an increased risk of placenta praevia. They require special obstetric attention during pregnancy and labour to manage the increased risk of bleeding complications and the foetal risk for prematurity. In pregnant women with complex EM (DIE; AM, rASRM III-IV), we recommend that placenta praevia be excluded by qualified ultrasound examination. Women with placenta praevia and DIE in the posterior compartment with known adhesions are at risk of bleeding complications. We recommend improving patients’ haemoglobin levels before delivery by following the guidelines for patient blood management [36].

To reduce the risk of abdominal wall EM, we suggest that the mode of delivery of every woman should be assessed by a specialist. Ideally, it should be possible to consult an endometriosis specialist with surgical experience.

Prospective studies with larger numbers of cases are needed to further evaluate the risk profile of women with AM and DIE during pregnancy and childbirth.

## Figures and Tables

**Table 1:** Demographic characteristics of case patients.

Age at EM Diagnosis in Years *Median (SD, Minimum–Maximum)*	28 (7.062, 17–47)
Age at case pregnancy in years *Median (SD, Minimum–Maximum)*	29.0 (5.024; 19–39)
Diagnosis EM or AM before occurrence of case pregnancy	63.4%
Surgery due to EM	100%
Surgery due to EM before the case pregnancy	61%

EM: endometriosis, SD: standard deviation, AM: adenomyosis.

**Table 2:** Diagnosis of EM in cases (n = 41).

	%
DIE (ENZIAN)	100%
AM	92.7%
rASRM stage	
rASRM I	4.9%
rASRM II	19.5%
rASRM III	19.5%
rASRM IV	53.7%
rASRM not classified	2.4%

DIE: deep infiltrating endometriosis, ENZIAN: classification for deep infiltrating endometriosis, AM: adenomyosis, rASRM: revised Classification of the American Society for Reproductive Medicine for endometriosis.

**Table 3:** Matching of age and mode of conception.

	Cases (n = 41)	Controls (n = 164)
Maternal age at end of pregnancy in years, median (SD; minimum–maximum).	29.0 (5024; 19–39)	29.5 (5117; 19–39)
Age over 34 at end of case pregnancy	9.8%	9.8%
Primipara	100%	100%
First pregnancy	92.7%	76.2%
Singleton pregnancy	100%	100%
IVF/ICSI	31.7%	31.7%

SD: standard deviation, IVF/ICSI IVF/ICSI: in vitro fertilisation/intracytoplasmatic sperm injection.

**Table 4:** Secondary endpoints: women with DIE compared to controls without EM.

	Cases (n = 41)	Controls (n = 164)	OR (95% CI)	*p*
Inpatient treatment during pregnancy	22.0	13.4	1815 (0.764–4.312)	0.172
Inpatient treatment in days median (SD; minimum–maximum)	5 (8594; 1–28) n = 9	3 (4598; 1–18) n = 22		0.453
Placenta praevia	9.8%	1.2%	8757 (1545–49,614)	0.004
Foetal presentation				
Vertex	92.7%	93.9%		
Breech	4.9%	5.5%		
Transverse or oblique	0%	0.6%		
Not specified	2.4%	0%		

OR: odds ratio, SD: standard deviation.

**Table 5:** Neonatal outcome.

	Cases (n = 41)	Controls (n = 164)	*p*
Live birth	100%	100%	
Umbilical cord pH < 7.10	0%, n = 35	3.1%, n = 163	0.294 °
5 min APGAR < 7	0%, n = 36	1.8%	0.414 °
10 min APGAR < 7	0%, n = 36	0%	
Birth weight in g, median (SD; minimum–maximum)	3330, (518.3. 1700–4070)	3322.5, (628.0. 620–4430)	0.868 *
Weight percentile median (SD; minimum–maximum)	41, (26.3; 2–94)	40, (27.9; 2–98)	0.986 *
Neonate with weight below 10th percentile	12.5%	9.1%	0.556 °
Neonate with weight over 95th percentile	0%	3.0%	0.258 °

APGAR: acronym for appearance, pulse, grimace, activation and respiration, classification for postnatal adaptation, SD: standard deviation, °: statistically not significant, *: statistically significant.

## Data Availability

The data that support the findings of this study are available on request from the corresponding author. The data are not publicly available due to privacy or ethical restrictions.

## Supplementary Materials

The following supporting information can be downloaded at: https://www.mdpi.com/article/10.3390/jcm13020414/s1. Data S1: Strobe statement; Data S2: inclusion and exclusion criteria in cases; Data S3: questionnaire; Data S4: exclusion criteria.
