# Supplementary material for: Women with Endometriosis—Who Is at Risk for Complications Associated with Pregnancy and Childbirth? A Retrospective Case–Control Study
Source: J Clin Med. 2024 Jan 11;13(2):414. doi: 10.3390/jcm13020414 (PMC10816776; doi:10.3390/jcm13020414)
Supplement: Supplementary file 1 [file jcm-13-00414-s001.zip › jcm-2760658-supplementary.pdf]

Supplementary data:

Data S1 Strobe statement

	Item No	Recommendation	Page
**Title and abstract**	1	(*a*) Indicate the study's design with a commonly used term in the title or the abstract	1
		(*b*) Provide in the abstract an informative and balanced summary of what was done and what was found	1
**Introduction**			
Background/rationale	2	Explain the scientific background and rationale for the investigation being reported	3-5
Objectives	3	State specific objectives, including any prespecified hypotheses	5
**Methods**			
Study design	4	Present key elements of study design early in the paper	5-6
Setting	5	Describe the setting, locations, and relevant dates, including periods of recruitment, exposure, follow-up, and data collection	5-6
Participants	6	(*a*) Give the eligibility criteria, and the sources and methods of case ascertainment and control selection. Give the rationale for the choice of cases and controls	5-6
		(*b*) For matched studies, give matching criteria and the number of controls per case	5-6
Variables	7	Clearly define all outcomes, exposures, predictors, potential confounders, and effect modifiers. Give diagnostic criteria, if applicable	5-6
Data sources/ measurement	8*	For each variable of interest, give sources of data and details of methods of assessment (measurement). Describe comparability of assessment methods if there is more than one group	5-6
Bias	9	Describe any efforts to address potential sources of bias	15, 18
Study size	10	Explain how the study size was arrived at	5-6
Quantitative variables	11	Explain how quantitative variables were handled in the analyses. If applicable, describe which groupings were chosen and why	5-6
Statistical methods	12	(*a*) Describe all statistical methods, including those used to control for confounding	5-6
		(*b*) Describe any methods used to examine subgroups and interactions	-
		(*c*) Explain how missing data were addressed	5-6
		(*d*) If applicable, explain how matching of cases and controls was addressed	5-6
		(*e*) Describe any sensitivity analyses	-
**Results**			
Participants	13*	(a) Report numbers of individuals at each stage of study - e.g. numbers potentially eligible, examined for eligibility, confirmed eligible, included in the study, completing follow-up, and analysed	7-8

	Item No	Recommendation	Page
		(b) Give reasons for non-participation at each stage	7-8
		(c) Consider use of a flow diagram	Figure 1
Descriptive data	14*	(a) Give characteristics of study participants (e.g. demographic, clinical, social) and information on exposures and potential confounders	7-8
		(b) Indicate number of participants with missing data for each variable of interest	7-8
Outcome data	15*	Report numbers in each exposure category, or summary measures of exposure	11-13
Main results	16	(*a*) Give unadjusted estimates and, if applicable, confounder-adjusted estimates and their precision (e.g., 95% confidence interval). Make clear which confounders were adjusted for and why they were included	11-13
		(*b*) Report category boundaries when continuous variables were categorized	
		(*c*) If relevant, consider translating estimates of relative risk into absolute risk for a meaningful time period	-
Other analyses	17	Report other analyses done—e.g. analyses of subgroups and interactions, and sensitivity analyses	-
**Discussion**			
Key results	18	Summarise key results with reference to study objectives	12
Limitations	19	Discuss limitations of the study, taking into account sources of potential bias or imprecision. Discuss both direction and magnitude of any potential bias	18-19
Interpretation	20	Give a cautious overall interpretation of results considering objectives, limitations, multiplicity of analyses, results from similar studies, and other relevant evidence	13-18
Generalisability	21	Discuss the generalisability (external validity) of the study results	13-18
**Other information**			
Funding	22	Give the source of funding and the role of the funders for the present study and, if applicable, for the original study on which the present article is based	20

Data S2 Inclusion and exclusion criteria in cases

Inclusion criteria
• At least one documented consultation with interview, clinical examination and transvaginal ultrasound with Prof. Dr. Mechsner in the period 01.01.2015 to 31.12.2019<br>• ENZIAN A, B, C or FI<br>• minimum age 18 years<br>• postal address in Germany stored in the clinic documentation programme SAP<br>• written consent to participate and return of completed questionnaire<br>• birth of at least one living or dead child with a birth weight of more than 500g
**Exclusion criteria**
• unclear diagnosis of DIE of the rectovaginal septum, vagina, sacrouterine ligaments, parametria, pelvic wall, ureters or intestines<br>• serious other maternal illness documented in the maternity record with initial diagnosis before the onset of the case pregnancy<br>• uterine surgery with opening of the uterine cavity before the onset of the case pregnancy<br>• multiple pregnancy

Data S3 Questionnaire

Outcome

1.
□ Lebendgeburt
□ Schwangerschaftsabbruch
□ Extrauteringravidität (EUG) bzw. Eileiterschwangerschaft
□ Fehlgeburt/Abort
□ Totgeburt

2.
□ Lebendgeburt
□ Schwangerschaftsabbruch
□ Extrauteringravidität (EUG) bzw. Eileiterschwangerschaft
□ Fehlgeburt/Abort
□ Totgeburt

3.
□ Lebendgeburt
□ Schwangerschaftsabbruch
□ Extrauteringravidität (EUG) bzw. Eileiterschwangerschaft
□ Fehlgeburt/Abort
□ Totgeburt

4.
□ Lebendgeburt
□ Schwangerschaftsabbruch
□ Extrauteringravidität (EUG) bzw. Eileiterschwangerschaft
□ Fehlgeburt/Abort
□ Totgeburt

5.
□ Lebendgeburt
□ Schwangerschaftsabbruch
□ Extrauteringravidität (EUG) bzw. Eileiterschwangerschaft
□ Fehlgeburt/Abort
□ Totgeburt

Allgemeine Krankheitsgeschichte

Wurden bei Ihnen Myome festgestellt bzw. leiden sie an einem sogenannten Uterus myomatosus?

In welchem Jahr wurde das zum ersten Mal festgestellt?
□ ja:
□ nein

Wurden Sie jemals wegen Adenomyose bzw. Endometriose operiert? Was wurde genau gemacht und in welchem Jahr? (Verwenden Sie ggf. die Rückseite für eine genauere Auflistung)
□ ja:
□ nein

Beratung und Betreuung in der Schwangerschaft und bei der Geburt
Wir möchten Sie dazu befragen, welche Rolle Adenomyose und tief-infiltrierende Endometriose in Gesprächen mit Ihren behandelnden Ärztinnen in der Schwangerschaftsvorsorge und in der Entbindungsklinik gespielt haben. Uns interessiert dabei auch ganz besonders Ihre persönliche Meinung.

Wurden Sie in der Schwangerschaft von Ihrer Frauenärztin auf Endometriose bzw. Adenomyose als Risikofaktor in der Schwangerschaft angesprochen?
□ ja □ nein

Wurden Sie in der Entbindungsklinik auf Endometriose bzw. Adenomyose als Risikofaktor in der Schwangerschaft angesprochen?
□ ja □ nein

Hätten Sie sich mehr Beratung zu Endometriose bzw. Adenomyose als Risikofaktor in der Schwangerschaft gewünscht…
… vor der ersten Schwangerschaft durch die Frauenärztin? □ ja □ nein
… in der Schwangerschaftsvorsorge? □ ja □ nein
… in der Entbindungsklinik? □ ja □ nein

Was hätten Sie sich zusätzlich an Beratung zum Thema Schwangerschaft bei Adenomyose und tief infiltrierender Endometriose gewünscht?

Verlauf und Outcome einzelner Schwangerschaften
Im folgenden möchten wir Ihnen Fragen zu der ersten Schwangerschaft stellen, bei der ein lebendes oder totes Kind mit mehr als 500g geboren wurde. Wir haben jeweils notiert, wo im Mutterpass die Antwort auf unsere Fragen vermerkt ist. Alternativ können Sie uns gerne Kopien aller beschriebenen Seiten im Mutterpass zusammen mit dem Fragebogen per Post oder als Fotos per Email an laura.ortlieb@charite.de schicken. Wir müssen Sie aber darauf hinweisen, dass der Versand von Daten über das allgemeine Internet nicht als sicher gilt.
Schwangerschaft 1
(1. Schwangerschaft, bei der ein lebendes oder totes Kind mit mehr als 500g geboren wurde)
Seite 5 bzw. 21 im Mutterpass: Anamnese und allgemeine Befunde / Erste Vorsorge-Untersuchung
Lagen Frühere eigene schwere Erkrankungen vor? Wenn ja, welche?
□ ja:
□ nein
Bestand Zustand nach Sterilitätsbehandlung?
(Wurde eine künstliche Befruchtung, also eine IVF oder ICSI durchgeführt?) □ ja □ nein
Bestand Zustand nach Sectio?
(Hatten Sie vor dieser Schwangerschaft schon einen Kaiserschnitt?) □ ja □ nein
Bestand Zustand nach anderen Uterusoperationen? Wenn ja, welchen?
□ ja:
□ nein
Seite 6 bzw. 22 im Mutterpass: Terminbestimmung
Berechneter Entbindungstermin
Berechneter Entbindungstermin (ggf. nach Verlauf korrigiert)
Bluthochdruck in der Schwangerschaft und Schwangerschaftsvergiftung
(Seiten 7 & 8 bzw. 23 & 24 im Mutterpass)

Kam es in dieser Schwangerschaft zu einem Schwangerschaftsbluthochdruck
(Gestationshypertonie)? □ ja □ nein
Kam es in dieser Schwangerschaft zu einer Schwangerschaftsvergiftung
(Präeklampsie)? □ ja □ nein
Schwangerschaft 1
(1. Schwangerschaft, bei der ein lebendes oder totes Kind mit mehr als 500g
geboren wurde)
Seite 9 bzw. 25 im Mutterpass: Stationäre Behandlung während der Schwangerschaft
Mussten Sie in dieser Schwangerschaft über Nacht im Krankenhaus bleiben?
von/bis
(wieviele Nächte?) Klinik Diagnose Therapie
Seite 15 bzw. 31 im Mutterpass: Abschluss-Untersuchung / Epikrise
Datum der Entbindung
Lebendgeburt □ ja □ nein
Einlinge oder
Mehrlinge
□ ein Kind □ Zwillinge □ Mehrlinge
Geburtsmodus □ vaginale Geburt □ Kaiserschnitt □ Zange oder
Saugglocke
(vaginal-operativ)
Kindslage □ Schädellage (SL) □ Beckenendlage (BEL) □ Querlage (QL)
Gewicht _ _ _ _ g
Apgar-Zahl 1'/5'/10' _ _ /_ _ /_ _
pH-Wert (Nabelarterie) _ ,_ _
Ist Ihr Kind in den ersten 7 Tagen nach Geburt verstorben? □ ja □ nein
Schwangerschaft 1
(1. Schwangerschaft, bei der ein lebendes oder totes Kind mit mehr als 500g
geboren wurde)
Wir möchten Ihnen nun noch ein paar Fragen stellen, die über die Informationen im Mutterpass hinausgehen. Wenn es für Sie möglich ist, können Sie uns auch gerne eine Kopie des Entlassungsbriefs aus der Entbindungsklinik per Post oder Email zukommen lassen. Wir müssen Sie aber darauf hinweisen, dass der Versand von Daten über das allgemeine Internet nicht als sicher gilt.
Beantworten Sie die folgenden Fragen bitte nur, wenn ein Kaiserschnitt durchgeführt wurde:
Warum wurde der Kaiserschnitt durchgeführt? (Was war die Indikation?)
□ nicht bekannt
Hatten Sie bereits Geburtswehen als der Kaiserschnitt durchgeführt wurde? □ ja □ nein
Beantworten Sie die folgenden Fragen bitte nur, wenn die Schwangerschaft vor 37+0
Schwangerschaftswochen endete:
Ist Ihnen der Grund für die Frühgeburt bekannt?
□ ja:
□ nicht bekannt
Wurde die Geburt medikamentös eingeleitet? □ ja □ nein
Wurde ein Kaiserschnitt durchgeführt? □ ja □ nein
Mutterkuchen
Lag in dieser Schwangerschaft eine Plazenta previa vor?
(Lag der Mutterkuchen vor dem inneren Muttermund?) □ ja □ nein
Blasensprung
Erfolgte der Blasensprung vor Ende der 37. Schwangerschaftswoche? □ ja □ nein
Erfolgte der Blasensprung vor Beginn der Geburtswehen? □ ja □ nein
Schwangerschaft 1
(1. Schwangerschaft, bei der ein lebendes oder totes Kind mit mehr als 500g
geboren wurde)
Kam es zu einer der folgenden sehr seltenen Komplikationen?
(Wenn möglich legen Sie eine Kopie vom Entlassungsbrief aus der Entbindungsklinik bei.)
spontane Uterusruptur in der Schwangerschaft
(Verletzung bzw. Riss der Gebärmutter vor Beginn der Geburtswehen) □ ja □ nein
Uterusruptur unter der Geburt
(Verletzung bzw. Riss der Gebärmutter nach Beginn der Geburtswehen) □ ja □ nein

spontane Darmperforation in der Schwangerschaft □ ja □ nein
spontanes Hämoperitoneum in der Schwangerschaft □ ja □ nein
Narbenendometriose im Bereich einer Kaiserschnittnarbe □ ja □ nein
andere schwere Komplikationen:
□ ja:
□ nein

Data S4 Exclusion criteria

- History of apoplexy, sinus vein thrombosis, intracerebral haemorrhage or myocardial infarction
- Arterial hypertension
- Autoimmune pancreatitis
- History of bariatric surgery
- Bleeding/thrombotic tendency or coagulation disorder
- C1 esterase deficiency
- Chronic inflammatory bowel disease, juvenile arthritis, rheumatoid arthritis, lupus erythematosus, psoriasis, Sjögren's syndrome
- Diabetes mellitus
- epilepsy
- First infection with CMV, toxoplasmosis or VZV during pregnancy
- Eating disorder
- Facioscapulohumeral muscular dystrophy
- Hydrocephalus with shunt, intracerebral vascular anomaly, hypoplasia of the pituitary gland, prolactinoma
- hyperthyreosis
- Infection with HIV, HBV, HCV, Treponema pallidum or tuberculosis
- History of complicated pelvic ring fracture
- Consanguinity
- Liver cirrhosis
- Wilson's disease
- Malignant hyperthermia
- History of malignancy
- Mucoviscidosis
- Multiple sclerosis
- Myasthenia gravis
- - Neurofibromatosis
- - Renal insufficiency, glomerulonephritis
- - History of organ transplantation
- - Proteus syndrome
- - Vaginal septum, uterus bicornis unicollis, uterus unicornis
- - Severe heart disease, defibrillator, pulmonary hypertension, valvular vitium
- - Sickle cell disease, thalassaemia
